# Mind Reading

**Published:** 1875-01

**Authors:** 


"MIND READING."—After our experiments with Brown in Music Hall, in New Haven, last week, we suggested to the audience that they could themselves do as well as Brown, and we that very night made experiments of a similar kind with two or three friends. We carried out the plan which he adopts, which is to put the back of the hand of the person operated on against the back of the head of the operator, holding it there permanently, and the other hand of the operator being touched lightly against the tips of the fingers of the hand of the person operated on. In this position the slightest tremor or agitation of the mind may be communicated through the tips of the fingers from one person to another. We found that in this way it was possible to detect the locality on which the mind of the person operated on was concentrated, provided he had his eyes upon it. About a quarter of our experiments were successful. These experiments were made in a small room and in a quiet way. Since that time we have made a different class of experiments to illustrate the remarkable results that are attained through *unconscious muscular action.* A lady with whom we are acquainted goes out of the room, and while she is absent an object is hidden. She returns, and two ladies who know where the object is stand up beside her in the middle of the room and place both of their hands upon her body, one hand in front, the other behind; all three stand there for a moment, the two women who know where the object is keeping their minds intensely concentrated on that locality. In a moment or so this lady who is to find the object moves off in the direction where it is, the other ladies with her still keeping their hands upon her, and in nearly all cases she finds it. This is accomplished by the unconscious muscular action of the two ladies who know where the object is, upon the person of the lady who is seeking it. This lady, who is quite successful in these experiments, thought it was necessary to hide keys, and supposed that "magnetism" had something to do with it. We told her that was not probable, and tried another object, and found that it made no difference what the object was. She supposed, also, that it was necessary that the object should be secreted on some person. We found that that was also not necessary. She does not always succeed in finding the exact locality at once, but she comes usually very near to it, and after hunting for it about as much as Brown, "the mind reader," usually does, she finds it. In some cases she goes directly to it. She very rarely fails.

In order to settle the question beyond dispute whether unconscious muscular action was the sole cause of this success in finding objects, we made the following crucial experiments with this lady: Ten letters of the alphabet were placed on a piano, the letters being written on large pieces of paper first. We directed her to see how many times she would get a letter which was in the mind of one of the observers in the room correct by chance purely, without any physical touch. She tried ten times and got it right twice. We then had her try ten experiments with the same letters in the same manner that Brown reads—that is, with the hand of the person operated on against the forehead of the operator, the hand of the operator lightly touching against the fingers of this hand, and the person operated on concentrating her mind all the while on the object, and looking at it. This is all there is of Brown's experiments and of those which imitate his. In ten experiments, tried this day, with the same letters, she was successful six times. We then tried the same number of experiments with a wire in the manner pursued by some of the faculty of Yale College and others. The wire was about ten feet long, and was so arranged—being made fast to a chair—that no unconscious muscular motion could be communicated through it from the person on whom she was operating to herself. She was successful but once out of ten times. Thus we see that by pure chance she was successful twice out of ten times; by utilizing unconscious muscular action in the method of Brown she was successful six times out of ten. When connected by a wire she was less successful than when she depended on pure chance without any physical connection. This seemed to us and to all who were co-operating with us a clear demonstration that unconscious muscular motion is the explanation of the success when connected in the method that Brown uses. In order still further to confirm this, we suggested to this lady to find objects with two persons touching her body in the manner we have above described. We told these two to deceive her, concentrating their minds on the object hidden, at the same time using conscious motion towards some other part of the room.

These experiments, several times repeated, showed that it was possible to deceive her, just as we had found it possible to deceive Brown in the same way at the Sturtevant House investigation. This lady, who is more uniformly successful than Brown, at least in these household experiments, is of a nervous constitution and susceptible, but others who try the same experiment meet with excellent success. Probably the majority of persons, with a little practice, can do all that Brown does or his imitators have done. Those who are successful with this method, and who carefully study the matter, tell us that they appreciate a slight thrill, as though a hair had touched their fingers, when they approach the locality of the hidden subject that is in the mind of the person operated on. Some are not aware of this at first until their attention is called to it, and they observe closely. In all these experiments, especial pains were taken by us to avoid the element of error that comes from unintentional collusion. Those who were in the room were ordered by us to keep perfectly still—not to laugh, speak, or move in their seats. Experiments that have been made in New Haven, Bridgeport, etc., lose their value partly because this element of error has not been guarded against, and partly because a fair comparison has not been made between what is accomplished when connected with a wire, or by the body, and when there is no connection, and no way of finding an object except by pure chance. The question may now, we think, be regarded as definitely settled as any question of this kind can be settled by rigid experimentation. These experiments can be confirmed without difficulty by any one who will take the same pains to avoid the errors that come first, from collusion; secondly, from unconscious muscular action; thirdly, from chance; and fourthly, from unintentional collusion of the audience. In comparing the success by pure chance with the success when there is unconscious muscular motion, the alphabet is a far better test than finding hidden objects, and it perfectly illustrates the whole principle. Mind-reading is really another form of the "learned pig" operations. In the case of the "learned pig," ("Educated Ben" excepted) the sign is given consciously by the master. In the case of "mind-reading," the sign is given unconsciously by the person operated on. * * *

We may say, in closing, that the notion entertained by some that these phenomena could be explained by "animal magnetism," is not only an unnecessary hypothesis, as shown by the above experiments, but is utterly opposed to what is known of electro-physiology.—*Archives of Electrology and Neurology.*

